# Safety and Efficacy of Losartan 50 mg in Reducing Blood Pressure among Patients with Post-Dialysis Euvolemic Hypertension: A Randomized Control Trial

**DOI:** 10.1038/s41598-017-17437-4

**Published:** 2017-12-18

**Authors:** Raja Ahsan Aftab, Amer Hayat Khan, Azreen Syazril Adnan, Syed Azhar Syed Sulaiman, Tahir Mehmood Khan

**Affiliations:** 1 Discipline of Clinical Pharmacy, School of Pharmaceutical Sciences Universiti Sains Malaysia, 11800 Gelugor, Penang Malaysia; 2 CKD Resource Centre, School of Medical Sciences, Universiti Sains Malaysia, Kubang Kerian, 16150 Kelantan Malaysia; 3 School of Pharmacy, Monash University Malaysia, Jalan Lagoon Selatan, Bandar Sunway, 45700 Selangor Malaysia; 4 Department of Pharmacy, Abasyn University, Peshawar, Pakistan

## Abstract

The aim of current study was to assess the effectiveness of losartan 50 mg in reducing blood pressure among post-dialysis euvolemic hypertensive patients, observing their survival trends and adverse events during the course of study. A multicentre, prospective, randomised, single-blind trial was conducted to assess the effect of losartan 50 mg every other day (EOD), once a morning (OM) among post-dialysis euvolemic hypertensive patients. Post-dialysis euvolemic assessment was done by a body composition monitor (BCM). Covariate Adaptive Randomization was used for allocation of participants to the standard or intervention arm. Of the total 229 patients, 96 (41.9%) were identified as post-dialysis euvolemic hypertensive. Final samples of 88 (40.1%) patients were randomized into standard and intervention arms. After follow-up of 12 months’ pre-dialysis systolic (p < 0.001) and diastolic (p 0.01), intradialysis diastolic (p 0.02), post-dialysis systolic (p < 0.001) and diastolic (p < 0.001) blood pressure was reduced from the baseline among intervention-arm patients Compared to only pre-dialysis systolic blood pressure (p 0.003) among standard arm patients after 12 months of follow. Total of six deaths were reported among standard-arm patients compared to 2 deaths among the intervention arm. Losartan 50 mg achieve an overall significant decline in blood pressure among post-dialysis euvolemic hypertensive patients.

## Introduction

The risk of mortality among end-stage renal disease (ESRD) patients receiving haemodialysis is 100 times greater than for the general population^1^. Every year, between 10–20% of patients on haemodialysis die, 45% of these deaths are attributed to cardiovascular events^2^. Net cardiovascular events in haemodialysis patients are multi-factorial, but elevated blood pressure in particular has a profound effect^3^. As the role of the kidney is impaired in end-stage renal disease patients, chronic volume overload of the renin-angiotensin-aldosterone system (RAAS), increased sympathetic activity and several other factors contribute to elevated blood pressure among haemodialysis patients. Despite known etiological causes, prevalence of hypertension among haemodialysis patients ranges between 60–80%^4^.

Exchangeable sodium, plasma volume and plasma-renin activity are almost twice as high in hypertensive haemodialysis patients as normotensive patients and are directly correlated with mean blood pressure^5,6^. This suggests that plasma volume and plasma-renin activity are two major factors contributing to hypertension among haemodialysis patients. A study on haemodialysis patients looking into response in RAAS with change in volume reported that patients with high plasma-renin activity before saline loading had an increase in blood pressure compared to normal or low plasma-renin activity^7^. Other studies have reported blood pressure to be more volume-dependent among haemolysis patients^8^. Textor *et al*. took this concept further and identified two groups of hypertensive haemodialysis patients: as volume-dependent and renin-dependent^9^.

Volume-dependent hypertensive haemodialysis patients can be managed by appropriate fluid removal. This is done by identifying the correct dry weight and extracting fluid accordingly. A body composition monitor (BCM) allows quantifiable analysis of excess extracellular volume through a comparison with a healthy population, thereby providing a reliable account of a patient’s dry weight and hydration status^10^. However, despite a reliable estimation of dry weight, and attaining post-dialysis euvolemic state, a group of patients are still hypertensive^11,12^, because of RAAS and are known as renin-dependent hypertensive patients^9^.

The current study was an attempt to analyse both concepts simultaneously, i.e accurately identifying patients’ dry weight, achieving euvolemic state post-dialysis, and treating post-dialysis euvolemic-but-hypertensive patients with an RAAS inhibitor. Thus the aim of current study was to assess the effectiveness of Losartan (ARB) in reducing blood pressure among post-dialysis euvolemic patients and assessing their survival trends.

## Method

The study protocol with complete research design has been published^12^. The study was a multicentre, prospective, randomised, parallel design, single-blind trial. The Hospital Universiti Sains Malaysia (HUSM) in Kelantan and its associated dialysis centres were the main research site. The study protocols were approved by the ethical and research committee of Universiti Sains Malaysia (USM/JEPeM/15050173), and the trial was registered under Australian New Zealand Clinical Trials Registry (ACTRN12615001322527, registered 02/12/2015). The study protocols met consolidated standard of reporting trials (CONSORT) guidelines. All study procedures were in accordance with clinical practice guidelines for haemodialysis from National Kidney Foundation Kidney Disease Outcome Quality Initiative (NKF KDOQI)^13^. The study purpose was explained and informed consent was obtained before enrolling study participants.

### Study participants

Post-dialysis euvolemic patients with systolic blood pressure >140 mmHg post-dialysis, patients aged 30–80, patients undergoing dialysis for of at least 12 months, and patients willing to participate were included in the trial. Patients with amputations, pre-existing heart disease, neoplasm and cystic kidneys, patients treated with ARBs and patients with symptomatic, predialytic hypotension (SBP less than 110 mm Hg) or high blood pressure >200/100 mmHg were excluded from the study.

### Study procedure

A body composition monitor device (BCM) made by Fresenius (BCM 4BJA3641) was used for assessing the volume status of patients at the end of a dialysis session and was also used for estimating patients’ dry weight^10,14^. After assessing dry weight, all efforts were made to ensure patients achieved their dry weight at the end of the dialysis sessions. Patients with systolic blood pressure >140/90 mmHg thirty minutes after the dialysis sessions underwent a volume assessment by a BCM device. The blood pressure (BP) readings were taken at sitting position by a haemodialysis staff nurse using a manually calibrated sphygmomanometer. Post-dialysis hypertensive patients (>140/90 mmHg) found euvolemic in three consecutive dialysis sessions were included in the trial. In addition, a regular BCM assessment for dry weight was done to ensure patients attained their specific dry weights and euvolemic states. Literature suggests that systolic blood pressure is constantly associated with cardiovascular adverse events^12,15^, which is why systolic blood pressure was considered as a marker for outcomes in the current trial.

### Randomization of study participants

Covariate-adaptive randomization was used for assigning all pre-screened participants who agreed to participate in the study to the intervention or standard arms^16^. On the basis of pre-existing literature, the covariates considered for the current study were age, gender, diabetes, and year of dialysis. All participants were randomized using covariate-adaptive randomization to prevent any influence of covariates on randomization^17^. Randomization of study participants was done by a computer program to minimize the risk of selection bias and avoid any influence of the researcher, the prescriber or the participant in the selection of group or medication^18^. Furthermore, all enrolled patients underwent a minimum two-week wash-out period to avoid any bias in the study.

### Rationale for intervention

An observational study suggests that an ARB in combination with other antihypertensive medication (but not an ACEI) may have a beneficial effect on cardiovascular mortality among ESRD patients on dialysis by reducing blood pressure^19^. Based on cost-effectiveness and availability, and the opinion of a panel of experts, a decision was taken to use an ARB (Losartan) for the intervention group.

### Standard and intervention study arms

The standard-arm patients received antihypertensive therapy (except RAAS inhibitors) including calcium channel blockers, diuretics, alpha and beta blockers, whereas the intervention-arm patients were prescribed ARB (losartan) alone or in combination with standard antihypertensive pharmacotherapy. In both study arms, upon discussion with a panel of experts, patients were allowed multiple antihypertensive pharmacotherapies to control blood pressure when necessary, to avoid any deterioration in a patient’s health. Study intervention (losartan) and antihypertensive medication was given to the patients on a weekly basis by the principle investigator. Only the study participants were blinded during the course of study.

### Study End Point

**Figure 1:**
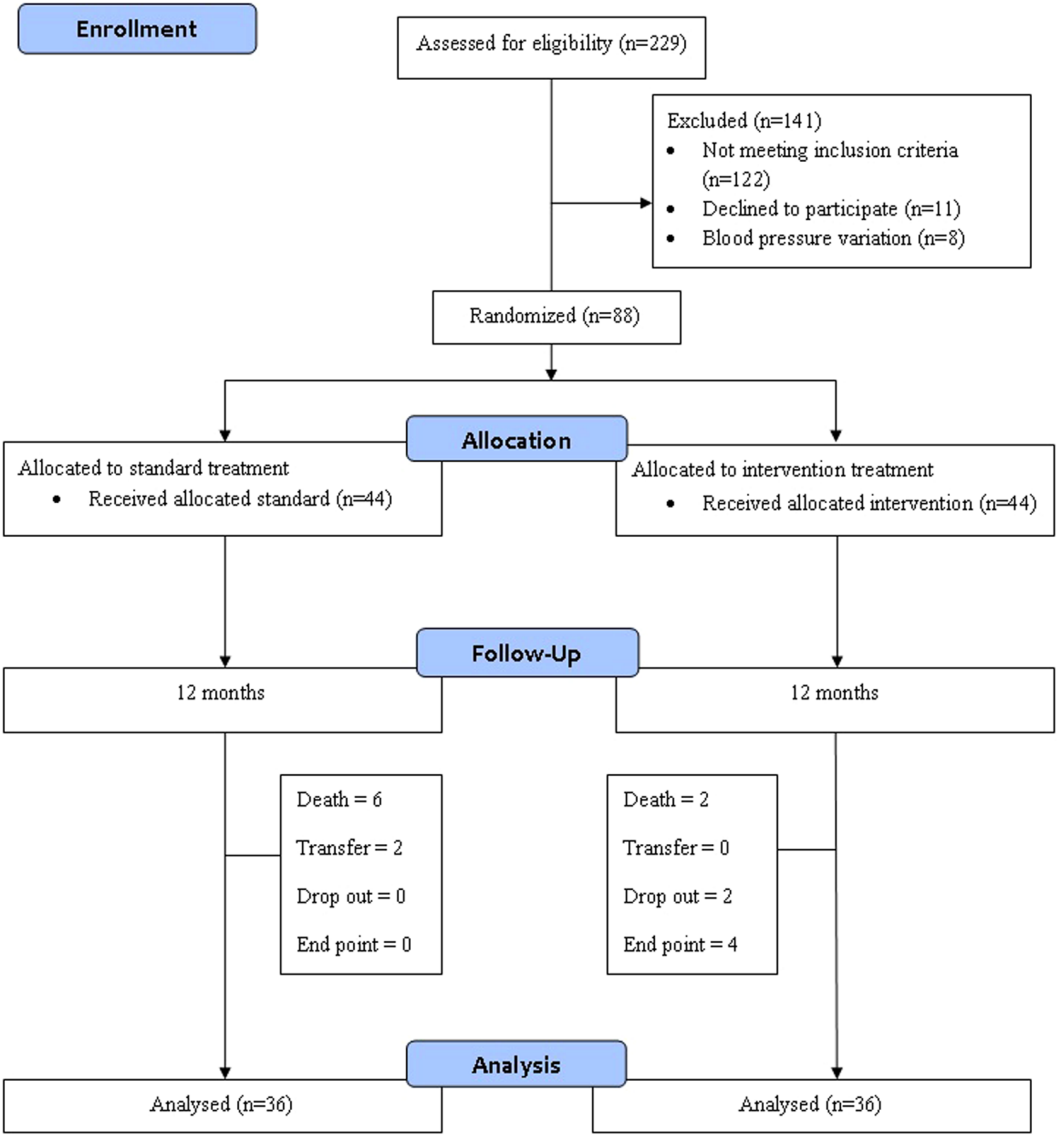
Study flow chart.

A medication dose of losartan 50 mg/day was initiated for three weeks as a test dose to note any incidence of hypotension. Upon satisfaction, patients were continued with 50 mg/day every day apart from dialysis days. A persistent > 180 mmHg post-dialysis blood pressure despite adding three hypertensive agents resulted in dose titration. The dose titration was initiated with losartan 50 mg to 100 mg followed by non-RAAS antihypertensive agents for the intervention group. For the standard group, the choice of dose titration was left to expert opinion^18,20^. The primary end point was achieving a post-dialysis blood pressure of <140/90 mmHg and maintaining this for four weeks, whereas the secondary end point was all causes of mortality. Figure 1 describes the study flow.

### Sample size and statistical analysis

The sample size for the current study was based on a statistical superiority trial (continuous data) design of a randomized control trial^21^.

$${\rm{N}}={\rm{2}}\times {(\frac{{z}_{1-\frac{a}{2}}+{z}_{1-\beta }}{\delta })}^{2}\times {{\rm{S}}}^{2}$$

Calculating the sample size using the equation above:

$$\begin{array}{c}{\rm{N}}=2\times {(\frac{1.96+0.845}{3})}^{2}\times 20.48\\ {\rm{N}}=35\end{array}$$

where, N = size per group; p = the response rate of standard treatment group; z = the standard normal deviate for a one or two sided x; δ = a clinically acceptable margin; S^2^ = Polled standard deviation of both comparison groups The sample size calculated from statistical superiority for randomized control trials is 35 for each arm, so altogether 70 euvolemic hypertensive patients. Moreover a 25% dropout was also included, making total of 88 patients (44 in each arm).

### Statistical analysis

Results were expressed as mean or percentage. Comparisons between treatment groups were made by using Wilcoxon tests after adjustment for the dynamic stratification variables (age, sex, years on dialysis, and diabetes). Cohen’s d test was applied to note the effect size. In addition, linear regression was applied to note any influence of patient characteristics on treatment outcome. This data is presented as hazard ratios and 95% confidence intervals. Statistical significance was set at *P* less than 0.05.

At the end of patient follow-up, both arms underwent survival analysis. Note that the patients that were dropouts/withdrew or transferred from the study could not be followed up. However, their final status at the end of patient follow-up was tracked. At the end of the trial, all transferred and withdrawn cases were alive and hence were categorised as living. Kaplan-Meier analyses were used for survival analysis after 12 months of patient follow-up. For this purpose, patient demographics and characteristics were analysed against patient status at the end of the trial through Kaplan-Meier analysis. All statistical calculations were performed using SPSS version 20.

## Results

A total of 229 haemodialysis patients underwent post-dialysis BCM analysis: 130 (56.8%) male and 99 (43.2%) female patients. The mean age of haemodialysis patients was 55.98 (SD ± 12) years. Since the study was carried out in the Malaysian state of Kelantan, the overwhelming majority of patients were of Malay ethnicity (n = 228, 99.6%). Of the total 229 patients, 113 (49.3%) had completed primary education and 117 (51.1%) were urban residents. 98 patients (42.8%) were categorised as normal weight, and 86 as overweight (37.6%), according to BMI classification. Thirty six (15.7%) patients were smokers and 2 (0.9%) patients were alcoholic. The largest number of patients (n = 89, 38.9%) were on dialysis for 2–3 years, followed by 1–2 years (n = 53, 23.1%), 3–4 years (n = 44, 19.4%), >5 years (n = 25, 10.9%) and 4–5 years on dialysis (n = 18, 7.9%). Hypertension (n = 186, 81.2%), anaemia (n = 176, 76.9%) and diabetes (n = 144, 62.9%) were the most common comorbidities among haemodialysis patients. A calcium channel blocker was the most prescribed antihypertensive among patients (n = 153, 66.8%) followed by a diuretic (n = 131, 62.9%) (Table 1).

**Table 1:** Patient demographic and clinical details.

Patient variables	N (%)
**Gender**	
Male	130 (56.8)
Female	99 (43.2)
**Age mean (±SD)**	55.98 (±12.0)
**Age group (years)**	
>30	7 (3.1)
31–40	19 (8.3)
41–50	37 (16.2)
>50	166 (72.5)
**Ethnicity**	
Malay	228 (99.6)
Indian	01 (0.4)
**Marital status**	
Single	6 (2.6)
Married	223 (97.4)
**Education**	
No formal education	48 (21)
Primary	113 (49.3)
Secondary	59 (25.8)
University/diploma	9 (3.8)
**BMI classification**	
Under weight	21 (9.2)
Normal range	98 (42.8)
Over weight	86 (37.6)
Obese	24 (10.5)
**Smoking status**	
Current smoker	36 (15.7)
Ex smoker	45 (19.7)
Never	148 (64.6)
**Alcohol status**	
Alcoholic	2 (0.9)
Ex-alcoholic	6 (2.6)
Non-alcoholic	221 (96.5)
**Resident**	
Urban	117 (51.1)
Rural	112 (48.9)
**Employment status**	
Employed	35 (15.3)
Un employed	45 (19.7)
Retired	73 (31.9)
House wife	73 (31.9)
Student	3 (1.3)
**Years of dialysis**	
1	53 (23.1)
2–3	89 (38.9)
3–4	44 (19.2)
4–5	18 (7.9)
>5	25 (10.9)
**Co-morbidities**	
Arthritis	17 (7.4)
Asthma	9 (3.9)
Hypertension	186 (81.2)
Diabetes	144 (62.9)
Anaemia	176 (76.9)
**Antihypertensive medication**	
Alpha blocker	43 (18.8)
Beta blocker	68 (29.7)
Calcium channel blocker	153 (66.8)
Angiotensin receptor blocker	11 (4.8)
Angiotensin converting enzyme inhibitors	2 (0.9)
Diuretic	131 (62.9)
Statins	144 (62.9)

**Figure 2:**
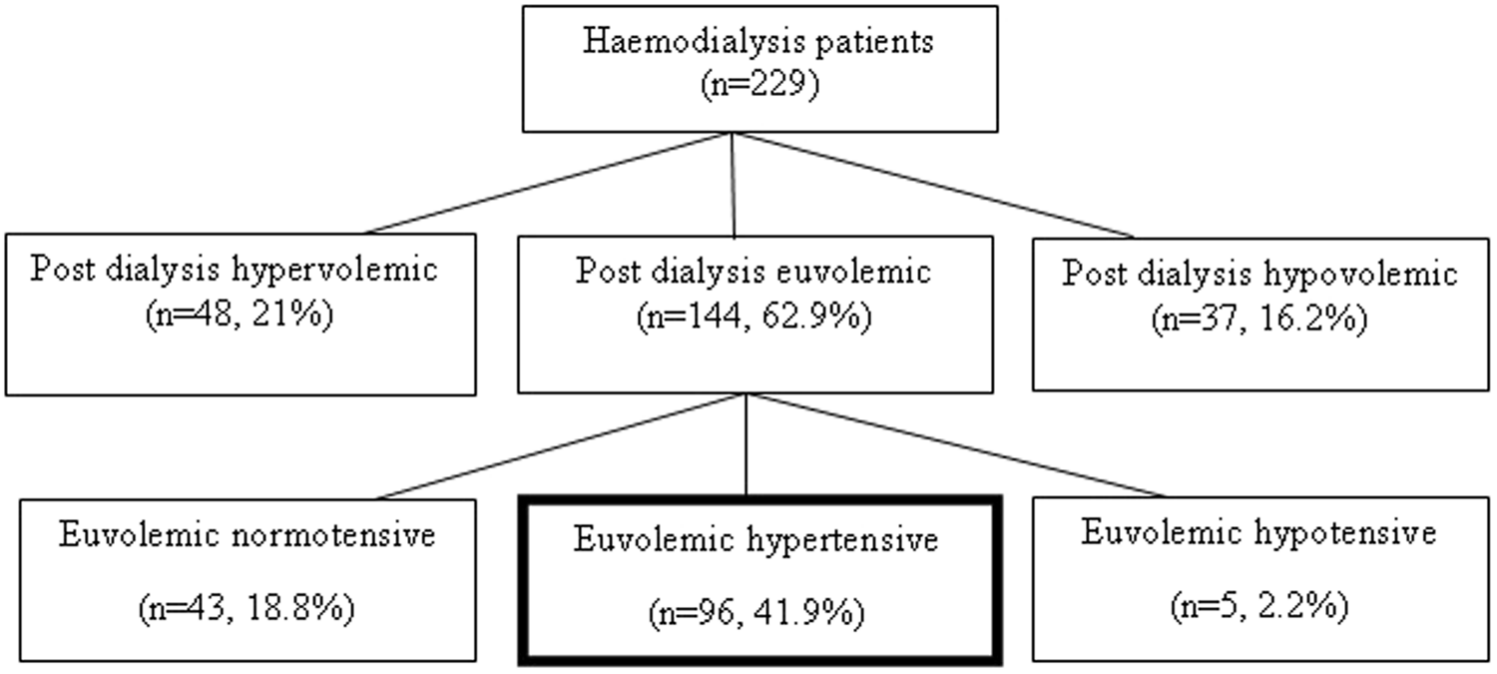
Selection of euvolemic hypertensive patients.

Of the total 229 haemodialysis patients, 48 (21%) were classified as post-dialysis hypervolemic, 37 (16.2%) were post-dialysis hypervolemic and 144 (62.9%) were post-dialysis euvolemic, according to post-dialysis BCM analysis. Furthermore, post-dialysis euvolemic patients (n = 144, 62.9%) were further classified with respect to their post-dialysis blood pressure. A total of 43 (18.8%) patients were euvolemic normotensive, 5 (2.2%) patients were euvolemic hypotensive whereas 96 (41.6) patients were classified as euvolemic hypertensive (Fig. 2).

Of the total 96 post-dialysis euvolemic patients, 8 (8.3%) patients experienced blood pressure variations and were thus dropped from the study. Altogether, final samples of 88 post-dialysis euvolemic patients were equally randomized into the standard and intervention arms. Twenty one (47.7%) male patients were randomized to the standard arm whereas 24 (54.5%) male patients were randomized to the intervention arm. Similarly, 23 (52.3%) female patients were randomized to the standard arm compared to 20 (45.4%) females in the intervention arm. The mean age of study participants was also comparable in the standard (54 ± 10.2 years) and intervention arms (53.7 ± 10.8 years). Thirty one (70.5%) diabetic patients were randomized to the standard arm, compared to 28 (63.6%) diabetic patients in the intervention arm. A calcium channel blocker was the most prescribed antihypertensive drug in both the study arms (standard n = 37, 84.1%; intervention = 32, 72.7%) (Table 2).

**Table 2:** Baseline Demographics and characteristics of study participants after randomization.

Variables	Standard N (%)	Intervention N (%)	P value*
**Gender**			
Male	21 (47.7)	24 (54.5)	0.59
Female	23 (52.3)	20 (45.5)	
**Age** (±**SD)**	54.0 (±10.2)	53.7 (±10.8)	
31–40	4 (9.1)	7 (15.9)	0.41
41–50	11 (25)	7 (15.9)	
>50	29 (65.9)	30 (68.2)	
**Comorbidities**			
Diabetes	31 (70.5)	28 (63.6)	0.54
**Years of dialysis**			
1	11 (25.0)	10 (22.7)	0.84
2–3	19 (43.2)	17 (38.6)	
3–4	8 (18.2)	8 (18.2)	
4–5	2 (4.5)	5 (11.4)	
>5	4 (9.1)	4 (9.1)	
**Education status**			
No formal education	3 (6.8)	11 (25)	**0.05**
Primary	31 (70.5)	22 (50)	
Secondary	8 (18.2)	10 (22.7)	
Territory	2 (4.5)	1 (2.3)	
**Socio-economic status**			
Low	5 (6.8)	15 (34.1)	**0.01**
Middle	31 (70.5)	23 (59.1)	
High	8 (18.2)	3 (6.8)	
**Smoking**			
Current smoker	11 (25)	9 (20.5)	0.05
Ex-smoker	2 (4.5)	10 (22.7)	
Never	31 (70.5)	25 (56.8)	
**Medication**			
Alpha Antagonist	12 (27.2)	7 (15.9)	0.28
Beta Antagonist	8 (18.1)	12 (27.2)	0.82
ARB	0	45 (100)	—
Ace Inhibitor	0	0	—
Calcium channel blocker	37 (84.1)	32 (72.7)	0.21
Diuritec	16 (36.3)	13 (29.5)	**0.01**
Statin	33 (75)	32 (71.7)	0.85
**Number of medications**			
1	9 (20.4)	1 (2.3)	**0.07**
2	14 (31.8)	11 (25)	
3	16 (36.3)	22 (50)	
>3	5 (11.3)	10 (22.7)	
Pre-dialysis systolic	167.5 (±18.2)	168.65 (±16.33)	—
Pre-dialysis diastolic	80.1 ( ± 12.7)	81.95 (±12.58)	—
Intradialysis systolic	153.6 (±20.6)	148.97 (±29.33)	—
Intradialysis diastolic	78.1 (±11)	81.20 (±19.72)	—
Post-dialysis systolic	157.5 (±14.3)	156.34 (±13.40)	—
Post-dialysis diastolic	80.6 (±12.6)	80.70 (±9.78)	—

Baseline pre-dialysis blood pressure among standard-arm patients was 167.5 mmHg (SD ± 18.2), which at the end of the 12-month follow-up was reduced to 162.4 mmHg (SD ± 10.2), giving a difference of 5.1 mmHg. This drop in pre-dialysis systolic blood pressure was statistically significant (Cohen’s d = 0.54, p 0.003). Whereas baseline pre-dialysis systolic blood pressure in the intervention arm was 168.65 mmHg (SD ± 16.33), at the end of the 12-month follow-up it was reported as 158.45(SD ± 10.65), giving a difference of −10.23 mmHg. Statistical analysis suggests a significant decline in pre-dialysis systolic blood pressure (p < 0.001) with a large effect size (Cohen’s d 0.94).

Post-dialysis systolic blood pressure among standard-arm patients was 157.5 (±14.3) mmHg, which at the end of the 12-month follow-up was reduced to 156.8 (±11.3) mmHg, giving a difference of 0.7 mmHg. This reduction is post-dialysis blood pressure was statistically non-significant (Cohen’s d 0.17, p 0.42) compared to baseline post-dialysis blood pressure that was 156.34 (SD ± 13.40) and at the end of the 12-month follow-up was 149.73 (SD ± 10.21), so a difference of 6.6 mmHg. This decline in post-dialysis systolic blood pressure was statistically significant (p < 0.001) with a large effect size (Cohen’s d 1.19) among intervention-arm patients (Table 3).

**Table 3:** Changes in baseline blood pressure reading and after 12 months of follow up.

Variable	Standard Baseline (±SD)	12^th^ month (±SD)	Cohen’s *d*	P value	Intervention Baseline (±SD)	12^th^ month (±SD)	Cohen’s *d*	P value
Interdialytic weight gain	1.6 (±0.6)	1.5 (±0.3)	—	—	1.94 (1.1)	1.49 (0.23)	—	—
Pre dialysis systolic	167.5 (±18.2)	162.4 (±10.2)	0.54	0.003	168.65 (16.33)	158.45 (10.65)	0.94	<0.001
Pre dialysis diastolic	80.1 (±12.7)	79.8 (±6.5)	0.12	0.68	81.95 (12.58)	76.57 (7.59)	0.45	0.01
Intradialysis systolic	153.6 (±20.6)	154.2 (±11.7)	0.017	0.63	148.97 (29.33)	151.66 (13.70)	0.01	0.25
Intradialysis diastolic	78.1 (±11)	78.8 (±7.6)	0.07	0.86	81.20 (19.72)	73.60 (5.39)	0.34	0.023
Post dialysis systolic	157.5 (±14.3)	156.8 (±11.3)	0.17	0.42	156.34 (13.40)	149.73 (10.21)	1.19	<0.001
Post dialysis diastolic	80.6 (±12.6)	80.3 (±5.5)	0.24	0.30	80.70 (9.78)	73.74 (7.04)	0.95	<0.001

Simple linear regression was applied for factors related to post-dialysis systolic blood pressure among both standard- and intervention-arm study participants. Analysis suggests that diabetes (standard p 0.03, intervention p 0.02) was related to post-dialysis systolic blood pressure among both study arm patients. Diabetes and ex-smokers (p 0.04) were statistically associated with post-dialysis blood pressure among standard arm patients. Similarly, male gender (p 0.02), patients that never smoked (0.04) and patients that were on diuretics (p 0.03) were statistically associated with post-dialysis blood pressure among intervention-arm patients (Table 4).

**Table 4:** Patient factors affecting post dialysis systolic blood pressure.

**Variables**	**Baseline N(%)**	**Standard**			**Intervention**			
		**Odd ratio**	**95%CI**	**P value**	**Baseline N(%)**	**Odd ratio**	**95%CI**	**P value**
**Gender**								
Male	21 (47.7)	0.03	−7.32, 8.62	0.85	24 (54.5)	0.38	−1.21, 14.21	**0.02**
Female	23 (52.3)	Reference			20 (45.5)	Reference		
**Age group**								
31–40	4 (9.1)	−0.11	−16.33,8.41	0.52	7 (15.9)	−0.01	−11.77, 10.55	0.91
41–50	11 (25)	0.18	−4.12,13.64	0.28	7 (15.9)	0.09	−6.89,11.86	0.59
>50	29 (65.9)	−0.09	−10.28,5.93	0.58	30 (68.2)	−0.06	−9.24,6.40	0.71
**Comorbidity**								
Diabetes	31 (70.5)	−0.36	−16.80, 0.98	**0.03**	28 (63.6)	−0.37	−15.25,−1.19	**0.02**
**Smoking**								
Current smoker	11 (25)	−0.17	−13.53,4.24	0.29	9 (20.5)	−0.25	−15.06,2.09	0.13
Ex-smoker	2 (4.5)	0.34	−0.73,32.84	**0.04**	10 (22.7)	−0.15	−13.52, 5.08	0.36
Never	31 (70.5)	−0.01	−8.54, 8.45	0.99	25 (56.8)	0.33	0.045,13.84	**0.04**
**Dialysis years**								
1	11 (25.0)	−0.05	−10.54,7.93	0.77		−0.18	−12.75,3.84	0.28
2–3	19 (43.2)				1 (2.3)	0.10	−4.95,9.03	0.55
3–4	8 (18.2)	−0.15	−8.60,7.94	0.93	11 (25)	0.21	−3.53,16.26	0.20
4–5	2 (4.5)	0.02	−9.42, 10.71	0.89	22 (50)	−0.03	−12.28,10.04	0.84
>5	4 (9.1)	0.08	−13.08, 21.30	0.62	10 (22.7)	−0.16	−22.48,7.72	0.32
**Medication**								
Alpha Antagonist	12 (27.2)	−0.13	−11.60,5.24	0.44	7 (15.9)	−0.04	−11.31,8.92	0.81
Beta Antagonist	8 (18.1)	0.19	−4.33,16.25	0.24	12 (27.2)	0.23	−2.17,12.63	0.16
Calcium channel blocker	37 (84.1)	0.08	−7.76,13.15	0.60	32 (72.7)	−0.21	−12.56,2.75	0.2
Diuritec	16 (36.3)	0.15	−4.23,11.45	0.35	13 (29.5)	0.35	−0.60,14.81	**0.03**

**Figure 3:**
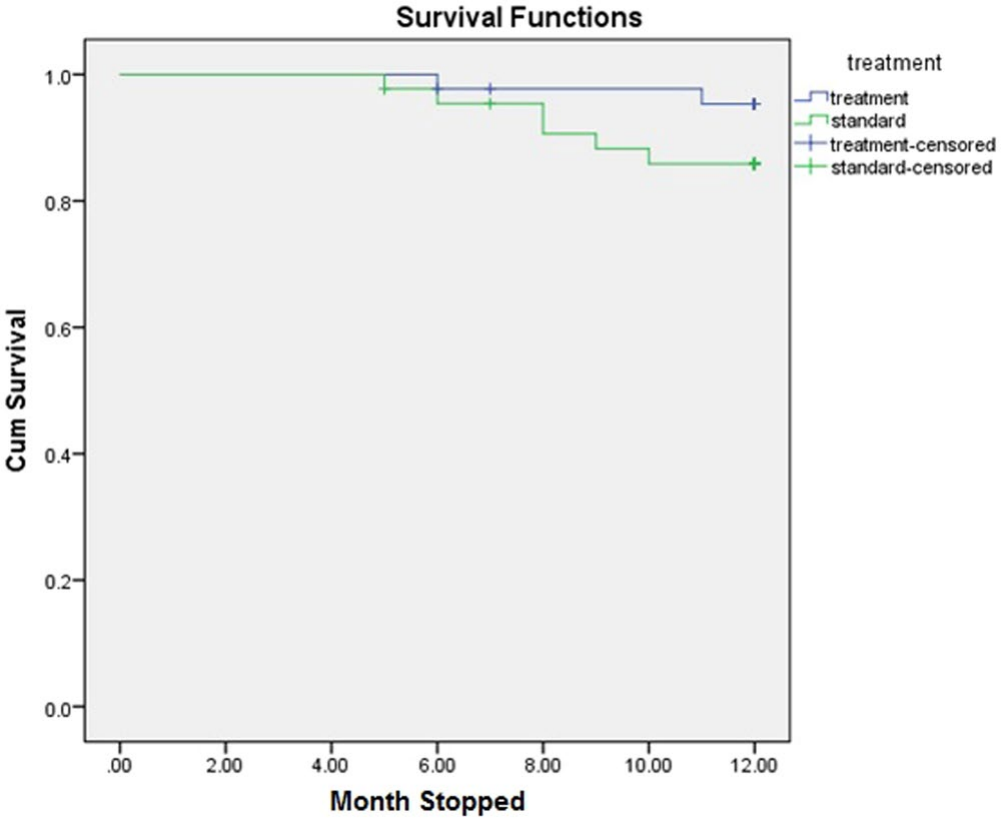
Study arm based survival analysis of study subjects.

**Figure 4:**
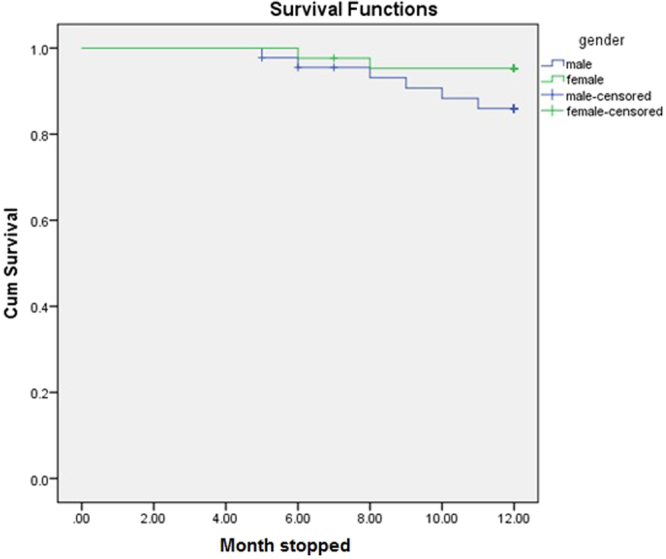
Gender based survival analysis of study subjects.

A total of 6 (13.6%) deaths were reported among patients in the standard arm compared to 2 (4.5%) deaths in the intervention arm. The probability of survival of standard-arm patients after 12 months of follow-up was 84% compared to 95% for intervention-arm patients (Fig. 3) However, Kaplan–Meier analysis indicates no significant difference in survival among patients in the standard or treatment arms. The majority of deaths were reported among male patients (n = 6, 13.6%) compared to 2 (4.5%) deaths among female patients. The probability of 1-year survival of men was 84% compared to 95% for women (Fig. 4). However, Kaplan-Meier analysis indicates no statistical difference in the survival of male and female study participants (Table 5).

**Table 5:** Survival trends of study participants through Kaplan-Meier analysis.

Kaplan-Meier analysis					
Variables	Death (N)	Mean	Standard error	95%CI	P value
**Trial arm**					
Standard	6	11.40	0.24	10.90,11.87	0.13
Treatment	2	11.61	0.13	11.57,12.10	
**Gender**					
Male	6	11.2	0.23	11.01,11.92	0.14
Female	2	11.7	0.16	11.44,12.08	
**Age group**					
31–40 years	1	11.18	0.54	10.43,12.47	0.95
41–50 years	2	11.38	0.38	10.74,12.25	
>50 years	5	11.61	0.14	11.38, 11.97	
**Smoking**					
Current smoker	2	11.65	0.29	11.06,11.22	0.97
Ex smoker	1	10.97	0.47	10.56,12.43	
Never	5	11.58	0.16	11.30, 11.96	
**Resident**					
Urban	4	11.48	0.24	11.02,11.96	0.92
Rural	4	11.56	0.13	11.49, 12.00	
**Diabetes**					
Yes	6	11.49	0.18	11.19, 11.91	0.67
No	2	11.55	0.21	11.33,12.16	
**Alpha antagonist**					0.51
Yes	1	11.68	0.21	11.35, 12.20	
No	7	11.46	0.17	11.23,11.91	
**Calcium channel blocker**					0.48
Yes	7	11.70	0.17	11.20,11.90	
No	1	11.93	0.15	11.54,12.14	
**Diuretics**					0.19
Yes	1	11.91	0.03	11.89,12.03	
No	7	11.33	0.29	11.03,11.85	

Adverse events were classified according to K/DOQI Clinical Practice Guidelines on Hypertension and Antihypertensive Agents in Chronic Kidney Disease. 2 patients in the intervention arm, so receiving losartan, suffered from mild hyperkalemia, as confirmed by the Naranjo scale. Similarly, 4 coughing, 3 dizziness and 1 dyspepsia cases were confirmed from Naranjo scale assessment (Table 6).

**Table 6:** Adverse events related to losartan during the course of trial.

Adverse events	Confirmed	Un decided
**Caused by inhibition ACE or blockade of AT1 receptor**		
Hypotension	—	4
Hyperkalemia	2	—
**Caused by inhibition of enzymes other than ACE or blockade of other receptor**		
Cough	4	—
Angioneurotic edema	—	—
**Allergic reactions**		
Skin rashes	—	—
Neutropenia, agranulocytosis	—	—
**Dysgeusia**		
Effect on the foetus	—	—
Kidney and lung toxicity	—	—
**Others**		
Dizziness	3	4
Diarrhea	—	2
Dyspepsia	1	—
URTI	—	—

## Discussion

The analysis of the current study suggests that blood volume and RAAS are two of the main factors contributing to hypertension among haemodialysis patients. Identifying, attaining ideal dry weight, and treating patients with RAAS inhibitors helps in reducing blood pressure significantly compared to non-RAAS antihypertensive therapy. The study findings are in line with preliminary findings that suggest that after 8 weeks of patient follow-up, intervention-arm patients were able to achieve a drop of 2.4 mmHg in post-dialysis systolic blood pressure compared to those in the standard arm (0.3 mmHg post-dialysis systolic blood pressure)^22^. After 12 months of patient follow-up, losartan 50 mg was able to achieve a difference of 6.6/6.9 mmHg from baseline post-dialysis blood pressure. Our analysis suggests this drop in post-dialyses blood pressure was significantly different (post-dialysis systolic blood pressure Cohen’s d 1.19, p < 0.001) from baseline and as compared to standard-arm patients that were treated with non-RAAS therapy (post-dialysis systolic Cohen’s d 0.17, p 0.42).

Not only was losartan 50 mg (OD) able to achieve a significant decline in post-dialysis blood pressure as compared to standard-arm patients, but patients in the intervention arm also observed a significant decline in pre-dialysis systolic blood pressure (Cohen’s d 0.94, p value < 0.001), pre-dialysis diastolic blood pressure (Cohen’s d 0.45, p 0.01), and intradialysis diastolic blood pressure (Cohen’s d 0.34, p 0.023). Perhaps the most vivid explanation in support of our findings with respect to the importance of volume control and RAAS was noticed among anephric patients, where both of the kidneys have been removed. These patients have no functioning RAAS and consequently have very low levels of aldosterone in their blood. Blood pressure in these patients is entirely dependent upon volume changes and is sensitive to changes in extra-cellular volume, with no response to changes in plasma-renin activity or hypotensive response to saralasin^23^.

Predictors for blood-pressure control among haemodialysis patients are multi-factorial. However, our analysis suggests that diabetes was the only patient factor that affected post-dialysis systolic blood pressure among both study arms. This finding was in agreement with previous literature that reported that higher mortality rates and uncontrolled blood pressure were common in diabetic patients on haemodialysis^24^. Moreover, the findings of the HELD trial also reported diabetes as a common predictor (both study arms) associated with post-dialysis high blood pressure. Possible explanation for diabetes as a factor for uncontrolled blood pressure among haemodialysis patients may include: diabetic patients have more comorbidities than non-diabetics; diabetic patients may not tolerate changes in systolic blood pressure as well as patients without diabetes; diabetes is associated with the aging process and patients with diabetes of a given age may experience higher mortality rates associated with changes in systolic blood pressure compared to older patients without diabetes^24^. Other factors that contributed towards post-dialysis systolic blood pressure were male gender and diuretics, among intervention-arm patients, compared to ex-smokers among standard-arm patients.

RAAS inhibitors are effective in lowering blood pressure and reducing cardiac events in non-haemodialysis patients^25^, thereby suggesting their role in managing blood pressure among haemodialysis patients. Bilateral nephrectomy was performed in patients with high levels of renin. However, with greater understanding of the blood-pressure mechanism in these renin-dependent individuals, bilateral nephrectomy was abandoned, as drugs that block RAAS activity were effective in controlling it, including ARBs, thereby suggesting they should be used. The results of the current study are an extension of these findings^26^.

Another important aspect often neglected is defining an optimal blood-pressure range among haemodialysis patients. National 4 kidney foundation guidelines recommend a pre-dialysis blood pressure of <140/90 mmHg and a post-dialysis blood pressure of <130/80 mmHg as targeted BPs for haemodialysis patients. However, there are some concerns regarding the targeted blood pressures, since most of the data is largely manipulated from observational studies from non-ESRD patients. Hence, targeted blood pressures among haemodialysis patients remain unclear^27^. Davenport *et al*. reported a higher incidence of intradialytic hypotension in patients achieving post-dialysis BP targets^28^. In turn, intradialytic hypotension is associated with mortality^29,30^, thereby raising questions regarding the recommended post-dialysis blood-pressure target range. Similarly, studies indicate a U-shaped or reverse J-shaped relationship between systolic blood pressure and mortality among ESRD patients^31,32^. Hence, a targeted post-dialysis of <140 mmHg systolic blood pressure was adopted for the current study.

A total of 88 post-dialysis euvolemic hypertensive patients were randomized into standard and intervention arms (44 each). 36 patients in each arm completed one year of follow-up. A total of 6 deaths and 2 transfer cases were reported among standard-arm patients, compared to 2 deaths, 2 drop-outs and 4 patients reaching end points among intervention-arm patients. None of the patients in the standard arm were able to achieve the study’s primary end point (post-dialysis systolic blood pressure <140 mmHg for four weeks). The 2 drop-outs reported in the intervention arm were due to hyperkalemia (>5.5 mEq/L).

Survival analysis among standard and intervention arms suggest higher mortality rates among standard-arm patients compared to intervention-arm patients, though the results are not statistically significant. Similarly, 75% of total deaths were reported in male patients whereas 62.5% of deaths were reported in patients aged >50 years, but neither of these variables were found to be statistically significantly associated with mortality rates after 1 year of follow-up. The literature suggests that the relationship between systolic blood pressure and mortality is not uniform across all age groups among the haemodialysis population, thereby suggesting optimal BP targets with different age groups^24^. In our study, the majority of mortalities were reported with patients >50 years old. This may be due to the presence of accelerated arthrosclerosis, which is common among haemodialysis patients. Previous studies have demonstrated that hypertension in haemodialysis patients is associated with increased mortality among patients that survive the first three years of dialysis^32,33^.

ARBs are usually well-tolerated; however hyperkalemia is frequently encountered among haemodialysis patients on RAAS inhibitors. Hence potassium levels and other blood parameters were closely tracked, to avoid any unwanted events. Two cases of hyperkalemia were reported in the current study, which resulted in study drop-outs. On the contrary, none of the study participants in the HELD trials reported any incidence of hyperkalemia^22^. Four cases of coughing, three cases of dizziness and one case of dyspepsia were recorded during the course of the study. However, they were treated and none of participants concerned dropped out.

## Limitations

A small sample size is one of the main limitations of this study. In addition, there could be some concerns about generalizing the results of this study, as the majority of participants were Malays, so the results cannot be generalized for the whole population of Malaysia. Keeping in mind the principle of genetic influence, it might be possible that other ethnic groups, i.e. Chinese or Indians, might have different outcomes to Malays. There is no consensus on an optimal blood-pressure goal among end-stage renal disease patients. Moreover, while an accurate BP measurement is fundamental to clinical practice and research, there is no consensus on which BP method is the best representation of actual blood pressure among haemodialysis patients, as studies indicate that routine pre-dialysis or post-dialysis BP readings may overestimate BP^34^. A further difference in measuring BP also exists, namely whether to take the BP reading in either the supine or sitting position, though the literature reported no difference between the two. In any case, there is no consensus on the optimal method of BP measurement among haemodialysis patients^35^.

## Conclusion

The current study indicates the importance of accurate assessment of clinical dry weight and treating post-dialysis euvolemic hypertensive patients with losartan, as this resulted in a significant decline in blood pressure. Inhibition of RAAS among post-dialysis euvolemic patients not only resulted in a significant decline in overall blood pressure but also reduced mortality compared to non-RAAS antihypertensive therapy. Reduction in overall blood pressure would not only lead to an increase in overall survival rates but may also improve the quality of life among ESRD patients.

### Ethical and Trial registration

The study protocols are approved from the Ethical and Research Committee of the Universiti Sains Malaysia (USM/JEPeM/15050173). The trial is registered under the Australia New Zealand Clinical Trial Registry (ACTRN12615001322527). The trial was registered on 2/12/2015 and the first patient was enrolled on 10/12/2015. The trial was formally initiated on 16/02/2016 and patients were followed up for 12 months.

## Electronic supplementary material

Consort 2010 checklist
